# Crystal structure of 3-mesityl-1-[(pyridin-2-yl)meth­yl]-3,4,5,6-tetra­hydro­pyrim­idin-1-ium bromide monohydrate

**DOI:** 10.1107/S2056989015003989

**Published:** 2015-03-04

**Authors:** Qi Quo, Liangru Yang, Pu Mao, Yongmei Xiao, Jinwei Yuan

**Affiliations:** a College of Chemistry and Chemical engineering, Henan University of Technology, Zhengzhou 450001, People’s Republic of China

**Keywords:** crystal structure, NHC precursor, tetra­hydro­pyrimidinium, hydrogen bonding

## Abstract

In the title hydrated salt, C_19_H_24_N_3_^+^·Br^−^·H_2_O, the values of the N—C bond lengths within the tetra­hydro­pyrimidinium ring indicate delocalization of the N=C double bond. In the cation, the dihedral angle formed by the pyridine and benzene rings is 14.97 (12)°. In the crystal, ions and water mol­ecules are linked by O—H⋯Br, O—H⋯N, C—H⋯Br and C—H⋯O hydrogen bonds into chains running parallel to the *b* axis.

## Related literature   

For background on the synthesis and properties of *N*-heterocyclic carbenes, see: Hopkinson *et al.* (2014 ▸); Mata *et al.* (2007 ▸); Dunsford & Cavell (2014 ▸); Mao *et al.* (2012 ▸).
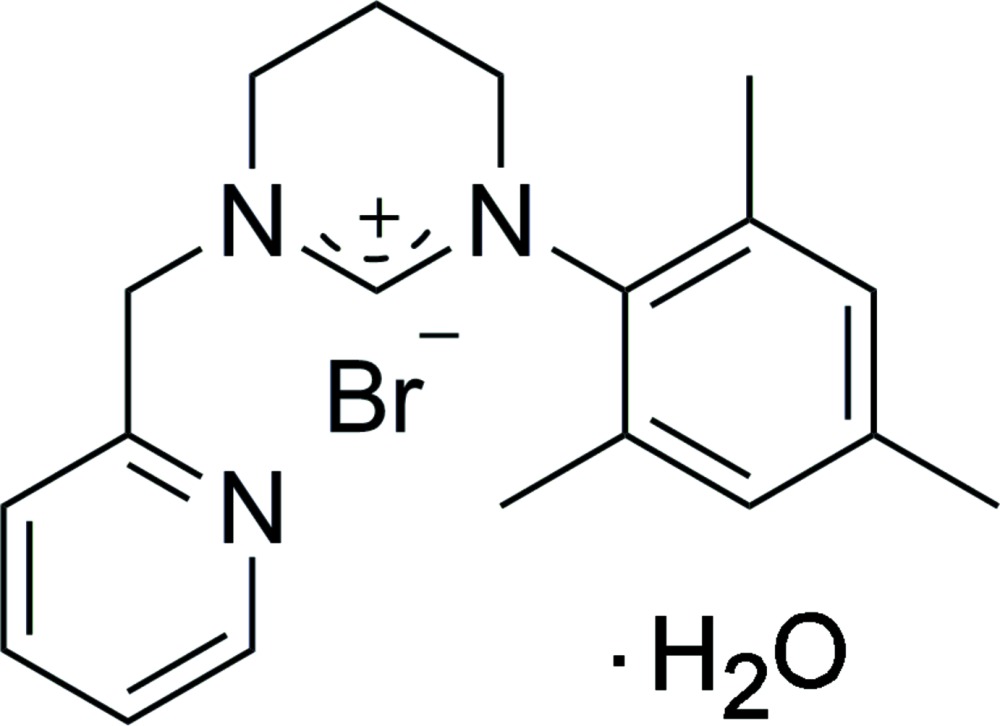


## Experimental   

### Crystal data  

C_19_H_24_N_3_^+^·Br^−^·H_2_O
*M* *_r_* = 392.34
Orthorhombic, 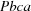

*a* = 15.5868 (5) Å
*b* = 14.6323 (4) Å
*c* = 17.0439 (6) Å
*V* = 3887.2 (2) Å^3^
*Z* = 8
Mo *K*α radiation
μ = 2.13 mm^−1^
*T* = 291 K
0.3 × 0.28 × 0.26 mm

### Data collection  

Agilent Xcalibur (Eos, Gemini) diffractometer
Absorption correction: multi-scan (*CrysAlis PRO*; Agilent, 2011 ▸) *T*_min_ = 0.910, *T*_max_ = 1.000
10196 measured reflections
3969 independent reflections
2522 reflections with *I* > 2σ(*I*)
*R*_int_ = 0.031

### Refinement  

*R*[*F* ^2^ > 2σ(*F* ^2^)] = 0.051
*wR*(*F* ^2^) = 0.134
*S* = 1.03
3969 reflections
227 parameters
1 restraint
H atoms treated by a mixture of independent and constrained refinement
Δρ_max_ = 0.83 e Å^−3^
Δρ_min_ = −0.81 e Å^−3^

### 

Data collection: *CrysAlis PRO* (Agilent, 2011 ▸); cell refinement: *CrysAlis PRO*; data reduction: *CrysAlis PRO*; program(s) used to solve structure: *SHELXS97* (Sheldrick, 2008 ▸); program(s) used to refine structure: *SHELXL97* (Sheldrick, 2008 ▸); molecular graphics: *OLEX2* (Dolomanov *et al.*, 2009 ▸); software used to prepare material for publication: *OLEX2*.

## Supplementary Material

Crystal structure: contains datablock(s) I, global. DOI: 10.1107/S2056989015003989/rz5148sup1.cif

Structure factors: contains datablock(s) I. DOI: 10.1107/S2056989015003989/rz5148Isup2.hkl

Supporting information file. DOI: 10.1107/S2056989015003989/rz5148Isup3.cml

**Fig. 1.**
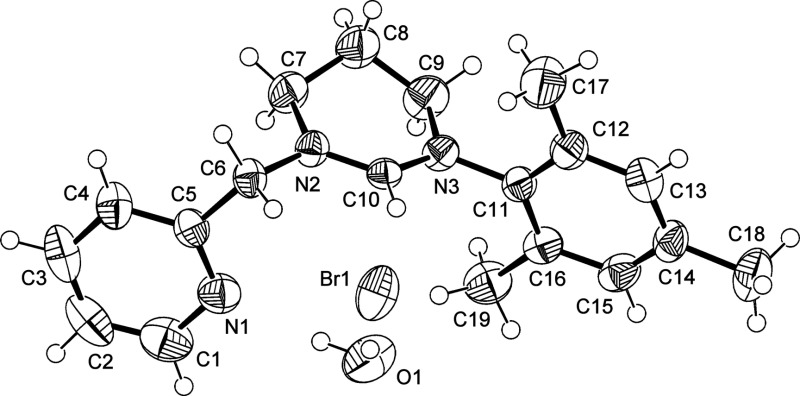
The molecular structure of the title compound showing 50% probability displacement ellipsoids.

. DOI: 10.1107/S2056989015003989/rz5148fig1.tif
The mol­ecular structure of the title compound showing 50% probability displacement ellipsoids.

CCDC reference: 1051286

Additional supporting information:  crystallographic information; 3D view; checkCIF report

## Figures and Tables

**Table 1:** Hydrogen-bond geometry (, )

*D*H*A*	*D*H	H*A*	*D* *A*	*D*H*A*
O1H1*A*Br1	0.85	2.46	3.292(4)	168
O1H1*B*N1	0.94(2)	1.94(3)	2.861(5)	165(7)
C6H6*B*Br1	0.97	2.87	3.815(3)	166
C3H3O1^i^	0.93	2.54	3.442(6)	165

Symmetry code: (i) 
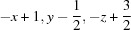
.

## supplementary crystallographic information

### S1. Comment

*N*-Heterocyclic carbenes (NHCs) have been widely used as ancillary ligands for the preparation of transition-metal based catalysts (Hopkinson *et al.*, 2014). Chelating NHC metal complexes have attracted particular research interest due to their enhanced stability and modular variability (Mata *et al.*, 2007). Most of the reported chelating NHC metal complexes contain NHC ligands based on imidazole-derived five-membered heterocyclic rings. Ring-expanded NHCs based on six-, seven-, or eight-membered heterocyclic rings possessing enhanced σ-donor ability and easy modular variability began to attract extensive attention in recent years (Dunsford & Cavell, 2014). It would be of interest to explore whether the introduction of ring expanded NHCs to a chelating framework will result in new chelating complexes displaying novel reactivity and enhanced catalytic activities. To the best of our knowledge, no report on chelating NHC metal complexes has been presented. Following our interest in the development of ring-expanded NHCs based on substituted 1,4,5,6-tetrahydropyrimidine and their metal complexes (Mao *et al.*, 2012), and with the intention of synthesizing chelating ring-expanded NHC metal complexes, we synthesized the chelating NHC precursor, 3-methyl-1-(pyridin-2-ylmethyl)-3,4,5,6-tetrahydropyrimidin-1-ium bromide and determined the structure of its monohydrate derivative. Research on the synthesis of carbene-metal complexes containing this ligand is currently in progress.

The molecular structure of the title compound is shown in Figure 1. As expected, the values of the bond distances within the pyrimidinyl ring indicate delocalization of the N═C bond that extends from N2 to N3 through C10, resulting in the increased acidity of the proton on C10 and convenient formation of a carbene functionality. In the cation, the benzene and pyridine rings form a dihedral angle of 14.97 (12)°. In the crystal structure, ions and water molecules are linked by O—H···N, O—H···Br, C—H···Br and C—H···O hydrogen bonds (Table 1) forming chains parallel to the *b* axis.

### S2. Experimental

A methanol solution (30 ml) of *N*-mesitylpropane-1,3-diamine (15 mmol, 2.88 g) was added dropwise to a methanol solution (30 ml) of pyridine-2-formaldehyde (15 mmol, 1.61 g) and the mixture was stirred at room temperature for 5 h. Infrared detection showed the disappearance of the carbonyl group. The mixture was then put into an ice-bath, and NaBH_4_ (120 mmol, 4.54 g) was added portion-wise for 1 h, before being warmed up to room temperature and then heated to 70 °C overnight. The solvent was evaporated and the residue was poured into a mixture of water (20 ml) and CH_2_Cl_2_ (20 ml). The resulting suspension liquid was filtered and the filtrate was extracted by CH_2_Cl_2_ (10 ml) for 3 times. The combined organic phase was evaporated and the residue obtained was dissolved in methanol (10 ml) for the following reaction directly. The solution was then treated with aqueous HCHO solution (36.5%, 15 mmol). The mixture was stirred at room temperature for 6 h before being evaporated. Purification of the residue by flash chromatography (silica, pentane/CH_2_Cl_2_/Et_3_N = 8/1/0.05, *v*/*v*/v) afforded the pure hexahydropyrimidine.

Hexahydropyrimidine (5 mmol, 1.48 g) was dissolved in DME (20 ml). NBS (5 mmol, 0.89 g) was added portion-wise and the resulting mixture was stirred at room temperature for 3 h, during which time a white precipitate formed. The precipitate was filtered and washed with DME. Crystallization of the precipitate from CH_2_Cl_2_/diethyl ether (1:1 *v*/*v*) afforded the title product as colourless crystals.

### S3. Refinement

The water H atoms could be located in a difference Fourier map, but only one of them (H1B) could be refined freely. The second H atom (H1A) was refined using a rigid-body approximation, with O—H constrained to be 0.9 Å, and with *U*_iso_(H) = 1.5 *U*_eq_(O). All other H atoms were placed geometrically and refined as riding, with C—H = 0.93–0.97 Å, and with *U*_iso_(H) = 1.2 *U*_eq_(C).

### Crystal data

**Table:**

C_19_H_24_N_3_^+^·Br^−^·H_2_O	*D*_x_ = 1.341 Mg m^−^^3^
*M**_r_* = 392.34	Mo *K*α radiation, λ = 0.7107 Å
Orthorhombic, *P**b**c**a*	Cell parameters from 1997 reflections
*a* = 15.5868 (5) Å	θ = 3.5–23.8°
*b* = 14.6323 (4) Å	µ = 2.13 mm^−^^1^
*c* = 17.0439 (6) Å	*T* = 291 K
*V* = 3887.2 (2) Å^3^	Block, colourless
*Z* = 8	0.3 × 0.28 × 0.26 mm
*F*(000) = 1632	

### Data collection

**Table:**

Agilent Xcalibur (Eos, Gemini) diffractometer	3969 independent reflections
Radiation source: Enhance (Mo) X-ray Source	2522 reflections with *I* > 2σ(*I*)
Graphite monochromator	*R*_int_ = 0.031
Detector resolution: 16.2312 pixels mm^-1^	θ_max_ = 26.4°, θ_min_ = 3.0°
ω scans	*h* = −10→19
Absorption correction: multi-scan (*CrysAlis PRO*; Agilent, 2011)	*k* = −17→18
*T*_min_ = 0.910, *T*_max_ = 1.000	*l* = −12→21
10196 measured reflections	

### Refinement

**Table:**

Refinement on *F*^2^	Primary atom site location: structure-invariant direct methods
Least-squares matrix: full	Secondary atom site location: difference Fourier map
*R*[*F*^2^ > 2σ(*F*^2^)] = 0.051	Hydrogen site location: inferred from neighbouring sites
*wR*(*F*^2^) = 0.134	H atoms treated by a mixture of independent and constrained refinement
*S* = 1.03	*w* = 1/[σ^2^(*F*_o_^2^) + (0.053*P*)^2^ + 3.4187*P*] where *P* = (*F*_o_^2^ + 2*F*_c_^2^)/3
3969 reflections	(Δ/σ)_max_ < 0.001
227 parameters	Δρ_max_ = 0.83 e Å^−^^3^
1 restraint	Δρ_min_ = −0.81 e Å^−^^3^

### Special details

**Table:**

Geometry. All e.s.d.'s (except the e.s.d. in the dihedral angle between two l.s. planes) are estimated using the full covariance matrix. The cell e.s.d.'s are taken into account individually in the estimation of e.s.d.'s in distances, angles and torsion angles; correlations between e.s.d.'s in cell parameters are only used when they are defined by crystal symmetry. An approximate (isotropic) treatment of cell e.s.d.'s is used for estimating e.s.d.'s involving l.s. planes.
Refinement. Refinement of *F*^2^ against ALL reflections. The weighted *R*-factor *wR* and goodness of fit *S* are based on *F*^2^, conventional *R*-factors *R* are based on *F*, with *F* set to zero for negative *F*^2^. The threshold expression of *F*^2^ > σ(*F*^2^) is used only for calculating *R*-factors(gt) *etc*. and is not relevant to the choice of reflections for refinement. *R*-factors based on *F*^2^ are statistically about twice as large as those based on *F*, and *R*- factors based on ALL data will be even larger.

### Fractional atomic coordinates and isotropic or equivalent isotropic displacement parameters (Å^2^)

**Table:**

	*x*	*y*	*z*	*U*_iso_*/*U*_eq_	
Br1	0.26231 (3)	0.64141 (3)	0.81482 (3)	0.06536 (19)	
O1	0.3743 (3)	0.6154 (2)	0.6526 (2)	0.0809 (10)	
H1A	0.3514	0.6282	0.6965	0.121*	
H1B	0.400 (4)	0.557 (2)	0.655 (4)	0.16 (3)*	
N1	0.4370 (2)	0.4358 (2)	0.68596 (18)	0.0522 (8)	
N2	0.25056 (17)	0.39948 (18)	0.67358 (16)	0.0366 (7)	
N3	0.17792 (18)	0.48218 (18)	0.57935 (16)	0.0388 (7)	
C1	0.5213 (3)	0.4180 (4)	0.6729 (3)	0.0662 (12)	
H1	0.5530	0.4597	0.6437	0.079*	
C2	0.5614 (3)	0.3423 (4)	0.7005 (3)	0.0711 (14)	
H2	0.6191	0.3325	0.6894	0.085*	
C3	0.5176 (3)	0.2814 (4)	0.7439 (3)	0.0701 (13)	
H3	0.5445	0.2294	0.7634	0.084*	
C4	0.4320 (2)	0.2977 (3)	0.7589 (2)	0.0543 (10)	
H4	0.4000	0.2566	0.7885	0.065*	
C5	0.3947 (2)	0.3756 (2)	0.7295 (2)	0.0402 (8)	
C6	0.3017 (2)	0.3970 (2)	0.7458 (2)	0.0398 (8)	
H6A	0.2783	0.3510	0.7808	0.048*	
H6B	0.2977	0.4557	0.7720	0.048*	
C7	0.2405 (2)	0.3121 (2)	0.6312 (2)	0.0515 (10)	
H7A	0.2329	0.2627	0.6686	0.062*	
H7B	0.2917	0.2997	0.6007	0.062*	
C8	0.1647 (3)	0.3168 (3)	0.5782 (3)	0.0603 (11)	
H8A	0.1651	0.2641	0.5437	0.072*	
H8B	0.1128	0.3141	0.6095	0.072*	
C9	0.1631 (3)	0.4013 (2)	0.5297 (2)	0.0590 (11)	
H9A	0.2072	0.3978	0.4897	0.071*	
H9B	0.1080	0.4067	0.5038	0.071*	
C10	0.21877 (19)	0.4758 (2)	0.64654 (18)	0.0331 (7)	
H10	0.2254	0.5285	0.6765	0.040*	
C11	0.1455 (2)	0.5698 (2)	0.55251 (19)	0.0374 (8)	
C12	0.0677 (2)	0.6013 (3)	0.5810 (2)	0.0464 (9)	
C13	0.0378 (2)	0.6844 (3)	0.5527 (2)	0.0513 (10)	
H13	−0.0137	0.7073	0.5718	0.062*	
C14	0.0822 (3)	0.7342 (2)	0.4968 (2)	0.0479 (9)	
C15	0.1587 (3)	0.6994 (2)	0.4690 (2)	0.0495 (9)	
H15	0.1887	0.7320	0.4310	0.059*	
C16	0.1922 (2)	0.6172 (2)	0.4960 (2)	0.0417 (8)	
C17	0.0167 (3)	0.5487 (3)	0.6414 (3)	0.0767 (14)	
H17A	0.0448	0.5527	0.6914	0.115*	
H17B	0.0128	0.4858	0.6258	0.115*	
H17C	−0.0399	0.5742	0.6454	0.115*	
C18	0.0496 (3)	0.8260 (3)	0.4680 (3)	0.0712 (13)	
H18A	0.0345	0.8634	0.5122	0.107*	
H18B	0.0000	0.8170	0.4356	0.107*	
H18C	0.0937	0.8558	0.4382	0.107*	
C19	0.2774 (3)	0.5823 (3)	0.4660 (2)	0.0587 (11)	
H19A	0.2681	0.5294	0.4337	0.088*	
H19B	0.3133	0.5661	0.5096	0.088*	
H19C	0.3049	0.6291	0.4356	0.088*	

### Atomic displacement parameters (Å^2^)

**Table:**

	*U*^11^	*U*^22^	*U*^33^	*U*^12^	*U*^13^	*U*^23^
Br1	0.0891 (4)	0.0542 (3)	0.0528 (3)	0.0202 (2)	0.0064 (2)	−0.0031 (2)
O1	0.092 (3)	0.062 (2)	0.089 (3)	−0.0072 (19)	0.012 (2)	0.009 (2)
N1	0.0478 (18)	0.0560 (19)	0.0527 (19)	−0.0023 (16)	0.0061 (16)	0.0012 (17)
N2	0.0404 (15)	0.0322 (14)	0.0371 (15)	0.0037 (12)	0.0002 (14)	−0.0037 (13)
N3	0.0463 (16)	0.0359 (15)	0.0343 (15)	−0.0024 (14)	−0.0022 (13)	−0.0015 (13)
C1	0.059 (3)	0.084 (3)	0.056 (3)	−0.010 (3)	0.012 (2)	−0.005 (2)
C2	0.039 (2)	0.108 (4)	0.066 (3)	0.008 (3)	−0.006 (2)	−0.013 (3)
C3	0.059 (3)	0.086 (3)	0.066 (3)	0.027 (3)	−0.019 (2)	−0.001 (3)
C4	0.054 (2)	0.060 (2)	0.049 (2)	0.012 (2)	−0.0039 (19)	0.007 (2)
C5	0.0402 (18)	0.046 (2)	0.0341 (18)	0.0022 (17)	−0.0031 (16)	−0.0017 (16)
C6	0.0403 (19)	0.0441 (19)	0.0350 (17)	0.0053 (16)	−0.0008 (16)	0.0034 (16)
C7	0.058 (2)	0.036 (2)	0.060 (2)	0.0042 (18)	−0.004 (2)	−0.0077 (19)
C8	0.072 (3)	0.041 (2)	0.067 (3)	−0.003 (2)	−0.014 (2)	−0.011 (2)
C9	0.080 (3)	0.050 (2)	0.047 (2)	−0.008 (2)	−0.016 (2)	−0.0093 (19)
C10	0.0310 (17)	0.0345 (17)	0.0338 (17)	−0.0030 (15)	0.0041 (15)	0.0003 (15)
C11	0.0417 (19)	0.0375 (18)	0.0331 (17)	−0.0023 (16)	−0.0048 (16)	0.0020 (16)
C12	0.048 (2)	0.049 (2)	0.042 (2)	−0.0007 (18)	−0.0003 (18)	0.0061 (18)
C13	0.048 (2)	0.057 (2)	0.050 (2)	0.008 (2)	−0.0052 (19)	−0.001 (2)
C14	0.063 (2)	0.042 (2)	0.0390 (19)	0.000 (2)	−0.0183 (19)	0.0036 (17)
C15	0.065 (3)	0.047 (2)	0.0355 (18)	−0.012 (2)	−0.0045 (19)	0.0067 (18)
C16	0.050 (2)	0.0429 (19)	0.0322 (18)	−0.0049 (18)	−0.0001 (17)	−0.0010 (16)
C17	0.063 (3)	0.084 (3)	0.083 (3)	0.013 (3)	0.027 (3)	0.029 (3)
C18	0.093 (3)	0.053 (2)	0.068 (3)	0.012 (2)	−0.019 (3)	0.009 (2)
C19	0.061 (2)	0.063 (2)	0.052 (2)	−0.005 (2)	0.015 (2)	0.002 (2)

### Geometric parameters (Å, º)

**Table:**

O1—H1A	0.8501	C8—H8B	0.9700
O1—H1B	0.94 (2)	C8—C9	1.486 (5)
N1—C1	1.357 (5)	C9—H9A	0.9700
N1—C5	1.327 (4)	C9—H9B	0.9700
N2—C6	1.467 (4)	C10—H10	0.9300
N2—C7	1.477 (4)	C11—C12	1.385 (5)
N2—C10	1.306 (4)	C11—C16	1.392 (5)
N3—C9	1.473 (4)	C12—C13	1.388 (5)
N3—C10	1.314 (4)	C12—C17	1.512 (5)
N3—C11	1.453 (4)	C13—H13	0.9300
C1—H1	0.9300	C13—C14	1.384 (5)
C1—C2	1.356 (7)	C14—C15	1.381 (5)
C2—H2	0.9300	C14—C18	1.518 (5)
C2—C3	1.344 (6)	C15—H15	0.9300
C3—H3	0.9300	C15—C16	1.390 (5)
C3—C4	1.380 (6)	C16—C19	1.512 (5)
C4—H4	0.9300	C17—H17A	0.9600
C4—C5	1.375 (5)	C17—H17B	0.9600
C5—C6	1.510 (5)	C17—H17C	0.9600
C6—H6A	0.9700	C18—H18A	0.9600
C6—H6B	0.9700	C18—H18B	0.9600
C7—H7A	0.9700	C18—H18C	0.9600
C7—H7B	0.9700	C19—H19A	0.9600
C7—C8	1.489 (5)	C19—H19B	0.9600
C8—H8A	0.9700	C19—H19C	0.9600
			
H1A—O1—H1B	109.5	N3—C9—H9B	109.6
C5—N1—C1	116.4 (4)	C8—C9—H9A	109.6
C6—N2—C7	116.5 (3)	C8—C9—H9B	109.6
C10—N2—C6	121.5 (3)	H9A—C9—H9B	108.1
C10—N2—C7	121.8 (3)	N2—C10—N3	123.5 (3)
C10—N3—C9	121.3 (3)	N2—C10—H10	118.2
C10—N3—C11	120.4 (3)	N3—C10—H10	118.2
C11—N3—C9	118.3 (3)	C12—C11—N3	119.2 (3)
N1—C1—H1	118.4	C12—C11—C16	122.3 (3)
C2—C1—N1	123.1 (4)	C16—C11—N3	118.4 (3)
C2—C1—H1	118.4	C11—C12—C13	117.6 (3)
C1—C2—H2	120.1	C11—C12—C17	122.0 (3)
C3—C2—C1	119.8 (4)	C13—C12—C17	120.4 (3)
C3—C2—H2	120.1	C12—C13—H13	118.9
C2—C3—H3	120.7	C14—C13—C12	122.2 (4)
C2—C3—C4	118.6 (4)	C14—C13—H13	118.9
C4—C3—H3	120.7	C13—C14—C18	121.4 (4)
C3—C4—H4	120.5	C15—C14—C13	118.2 (3)
C5—C4—C3	119.0 (4)	C15—C14—C18	120.3 (4)
C5—C4—H4	120.5	C14—C15—H15	119.0
N1—C5—C4	123.0 (3)	C14—C15—C16	122.1 (3)
N1—C5—C6	116.3 (3)	C16—C15—H15	119.0
C4—C5—C6	120.7 (3)	C11—C16—C19	121.7 (3)
N2—C6—C5	111.8 (3)	C15—C16—C11	117.6 (3)
N2—C6—H6A	109.3	C15—C16—C19	120.8 (3)
N2—C6—H6B	109.3	C12—C17—H17A	109.5
C5—C6—H6A	109.3	C12—C17—H17B	109.5
C5—C6—H6B	109.3	C12—C17—H17C	109.5
H6A—C6—H6B	107.9	H17A—C17—H17B	109.5
N2—C7—H7A	109.7	H17A—C17—H17C	109.5
N2—C7—H7B	109.7	H17B—C17—H17C	109.5
N2—C7—C8	109.9 (3)	C14—C18—H18A	109.5
H7A—C7—H7B	108.2	C14—C18—H18B	109.5
C8—C7—H7A	109.7	C14—C18—H18C	109.5
C8—C7—H7B	109.7	H18A—C18—H18B	109.5
C7—C8—H8A	109.0	H18A—C18—H18C	109.5
C7—C8—H8B	109.0	H18B—C18—H18C	109.5
H8A—C8—H8B	107.8	C16—C19—H19A	109.5
C9—C8—C7	112.9 (3)	C16—C19—H19B	109.5
C9—C8—H8A	109.0	C16—C19—H19C	109.5
C9—C8—H8B	109.0	H19A—C19—H19B	109.5
N3—C9—C8	110.3 (3)	H19A—C19—H19C	109.5
N3—C9—H9A	109.6	H19B—C19—H19C	109.5
			
N1—C1—C2—C3	−1.0 (7)	C9—N3—C11—C12	97.5 (4)
N1—C5—C6—N2	62.8 (4)	C9—N3—C11—C16	−79.5 (4)
N2—C7—C8—C9	48.0 (5)	C10—N2—C6—C5	−110.9 (3)
N3—C11—C12—C13	−178.5 (3)	C10—N2—C7—C8	−23.0 (5)
N3—C11—C12—C17	2.1 (5)	C10—N3—C9—C8	24.2 (5)
N3—C11—C16—C15	177.8 (3)	C10—N3—C11—C12	−82.0 (4)
N3—C11—C16—C19	−3.6 (5)	C10—N3—C11—C16	101.0 (4)
C1—N1—C5—C4	−1.5 (5)	C11—N3—C9—C8	−155.3 (3)
C1—N1—C5—C6	178.3 (3)	C11—N3—C10—N2	−179.0 (3)
C1—C2—C3—C4	0.4 (7)	C11—C12—C13—C14	1.2 (5)
C2—C3—C4—C5	−0.4 (6)	C12—C11—C16—C15	0.9 (5)
C3—C4—C5—N1	1.0 (6)	C12—C11—C16—C19	179.5 (3)
C3—C4—C5—C6	−178.8 (4)	C12—C13—C14—C15	−0.1 (5)
C4—C5—C6—N2	−117.3 (4)	C12—C13—C14—C18	−178.4 (4)
C5—N1—C1—C2	1.5 (6)	C13—C14—C15—C16	−0.8 (5)
C6—N2—C7—C8	159.8 (3)	C14—C15—C16—C11	0.4 (5)
C6—N2—C10—N3	175.0 (3)	C14—C15—C16—C19	−178.3 (3)
C7—N2—C6—C5	66.3 (4)	C16—C11—C12—C13	−1.6 (5)
C7—N2—C10—N3	−2.1 (5)	C16—C11—C12—C17	179.0 (4)
C7—C8—C9—N3	−48.7 (5)	C17—C12—C13—C14	−179.3 (4)
C9—N3—C10—N2	1.5 (5)	C18—C14—C15—C16	177.6 (3)

### Hydrogen-bond geometry (Å, º)

**Table:**

*D*—H···*A*	*D*—H	H···*A*	*D*···*A*	*D*—H···*A*
O1—H1*A*···Br1	0.85	2.46	3.292 (4)	168
O1—H1*B*···N1	0.94 (2)	1.94 (3)	2.861 (5)	165 (7)
C6—H6*B*···Br1	0.97	2.87	3.815 (3)	166
C3—H3···O1^i^	0.93	2.54	3.442 (6)	165

Symmetry code: (i) −*x*+1, *y*−1/2, −*z*+3/2.
