# Supplementary figures and images for: Crystal structure of 3-mesityl-1-[(pyridin-2-yl)meth­yl]-3,4,5,6-tetra­hydro­pyrim­idin-1-ium bromide monohydrate
Source: Acta Crystallogr E Crystallogr Commun. 2015 Mar 4;71(Pt 4):o224. doi: 10.1107/S2056989015003989 (PMC4438856; doi:10.1107/S2056989015003989)

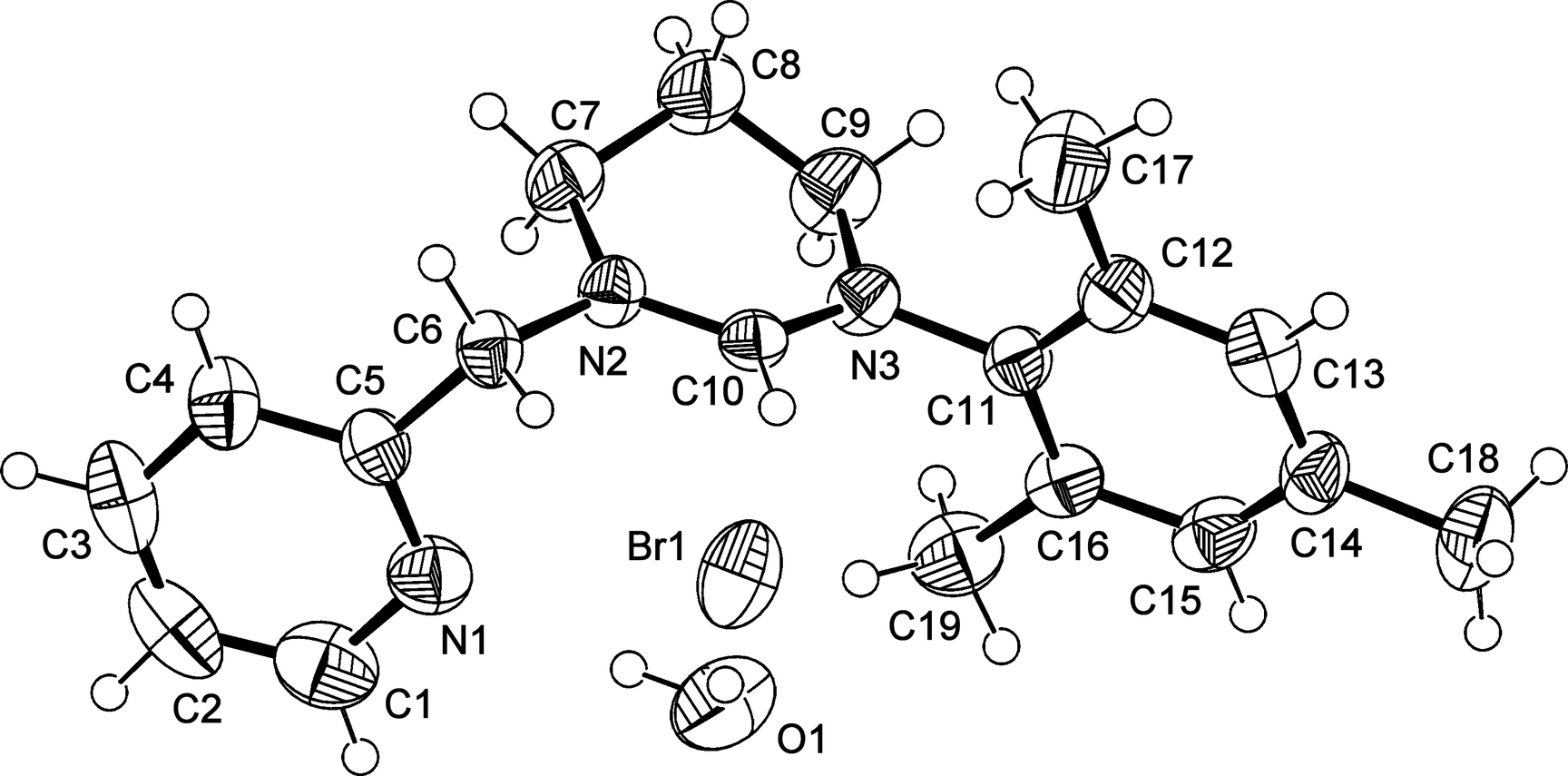

Supplement: Supplementary file 4 [file e-71-0o224-fig1.tif]
